# Are oral health behaviors associated with metabolic syndrome in the Azar cohort population?

**DOI:** 10.1186/s12903-023-03003-0

**Published:** 2023-06-08

**Authors:** MohammadAmin Tarighat Esfanjani, Neda Gilani, Ali Tarighat Esfanjani, Amir Mohammad Nourizadeh, Elnaz Faramarzi, Somayeh Hekmatfar

**Affiliations:** 1 Faculty of Dentistry, Ardabil University of Medical Sciences, Ardabil, Iran; 2 Statistics and Epidemiology department, Tabriz University of Medical Sciences, Tabriz, East Azerbaijan Iran; 3 Department of Clinical Nutrition, School of Nutrition and Food Sciences, Tabriz University of Medical Sciences, Tabriz, Iran; 4 Liver and Gastrointestinal Diseases Research center, Tabriz University of Medical Sciences, Tabriz, 51683343 Iran; 5 Department of Pediatric Dentistry, School of Dentistry, Ardabil University of Medical Sciences, Ardabil, Iran

**Keywords:** Oral health behaviors, Metabolic syndrome, Toothbrush, Cohort study

## Abstract

**Objective:**

Considering the rising prevalence of metabolic syndrome (MetS), this study aimed to investigate the relationship between MetS and its components with oral and dental health in the adult population of the Azar cohort.

**Methods:**

In this cross-sectional study oral health care behaviors, DMFT (decayed, missing, and filled teeth) index, and demographic data related to 15,006 patients (5112 in the MetS group and 9894 in the healthy group) of the Azar Cohort population aging from 35 to 70 were collected using appropriate questionnaires. The definition of MetS was based on the National Cholesterol Education Program Adult Treatment Panel III (ATP III) criteria. Then, the risk factors of MetS related to oral health behaviors were determined by proper statistical analysis.

**Results:**

The majority of MetS patients were female (66%) and uneducated (23%) (P < 0.001). In the MetS group, the DMFT index (22.15 ± 8.89) was significantly (*p* < 0.001) higher (20.81 ± 8.94) than the no MetS group. Not brushing at all was associated with increased odds of MetS (unadjusted OR = 1.12, adjusted OR = 1.18). Flossing less than once a day was associated with increased odds of abdominal obesity (unadjusted OR = 1.17, 95%CI = 1.03–1.32) and hyperglycemia (unadjusted OR = 1.88, 95%CI = 1.61–2.20).

**Conclusions:**

This study showed that in MetS patients of the Azar cohort study, oral hygiene was worse compared to that in the no MetS group. Further studies are suggested to encourage oral hygiene among the general population which has more beneficiary effects than has been known before.

## Introduction

Oral and dental infections are among the most common public health problems in most societies. The prevalence of dental caries and edentulism in the world’s adult population is 35% and 30%, respectively. In the Iranian people, the prevalence of caries of the first molar in children in the first years of school was 17.9%, and the mean decayed tooth and edentulous index were 2.85 ± 1.7, and 1.15 ± 1.84 respectively. The mean number of filled teeth is reported to be 3.33 ± 1.7 [1–3]. It is thought that chronic oral diseases such as periodontitis increase the risk of cardiovascular disease, diabetes, and metabolic syndrome (MetS); [4–6] for instance, several studies showed that the DFMT (decayed, missing, and filled teeth) index was significantly higher in patients who were overweight or obese compared to that in normal weighted subjects. Previous data suggest that patients with MetS should be regularly screened for periodontal diseases and pay special attention to their oral hygiene [7–9].

The prevalence of metabolic syndrome (MetS), also called X syndrome, is 20 to 46% in various studies, and the prevalence of this syndrome in Iran is reported to be 26% [10–12]. This syndrome consists of chronic inflammation and insulin resistance, which lead to an increased risk of heart disease, diabetes, stroke, and other disabilities. In recent years, with the changes in lifestyle, nutrition, and increased life expectancy, obesity and MetS have dramatically increased; therefore, MetS is a global pandemic [13].

As mentioned before, chronic oral diseases such as periodontitis increase the likelihood of MetS, and the cause is thought to be chronic inflammation due to the immune system responding to the bacteria in dental plaques leading to the alveolar bone and gingival degeneration [14]. Studies have shown that mild chronic inflammation is a risk factor for MetS [15]. Some studies have explored the presence of inflammation with oral microflora. Si et al., in patients with MetS; the most common oral microorganisms were *Catella granuloma* and *Neisseria*. In contrast, in the general population most common oral microorganism is *Peptococcus* [16]. This situation can accelerate the processes of MetS by creating a chronic inflammatory state [16].

On the other hand, some researchers suggested that the association between MetS and oral health could be attributed to factors that can cause both conditions, such as bad eating habits, and could not identify any correlation between oral health and MetS [17, 18].

It is thought that brushing and flossing reduce plaque load and immune response [19]. Some studies suggest that dental treatments can help lower the blood sugar levels in no MetS patients with diabetes. In return, drugs used in treating dyslipidemia, such as statins, may have beneficial and promising effects on several aspects of dental diseases. Some studies have reported the interference of drugs used in treating dyslipidemia with dental problems [20].

Socioeconomic inequalities have a close relationship with oral health. In a study by Hamasha et al., it was determined that in people with a higher body mass index, low education level, an underlying systemic disease, smoking, and edentulism, the DMFT index was significantly higher than in healthy subjects [21].

Due to the high costs of dental care services, most people do not have the opportunity to have regular dental checkups. On the other hand, awareness about oral and dental health behaviors is not at a favorable level. Despite the studies conducted in this field, very few studies have investigated the relationship between oral hygiene with MetS and its components. Therefore, this study aimed to investigate the relationship between oral health behaviors with each component of MetS in the adult population of the Azar cohort.

## Methods

### Study design & data sources

This cross-sectional study was approved by the regional committee of biomedical research (No: IR.ARUMS.REC.1401.046). The data was collected from the Azar cohort study, which is part of a large-scale Persian cohort study (Prospective Epidemiological Research Studies of Iranian Adults) [22].

### Setting & participants

From the 33,000 population (35–70 years) living in a region in the Northwest of Iran, 15,006 subjects aged 35 to 70 years were included in the Azar cohort. Azar cohort study is described in extensive detail elsewhere [23].

### Characteristics of the participants

**Fig. 1:**
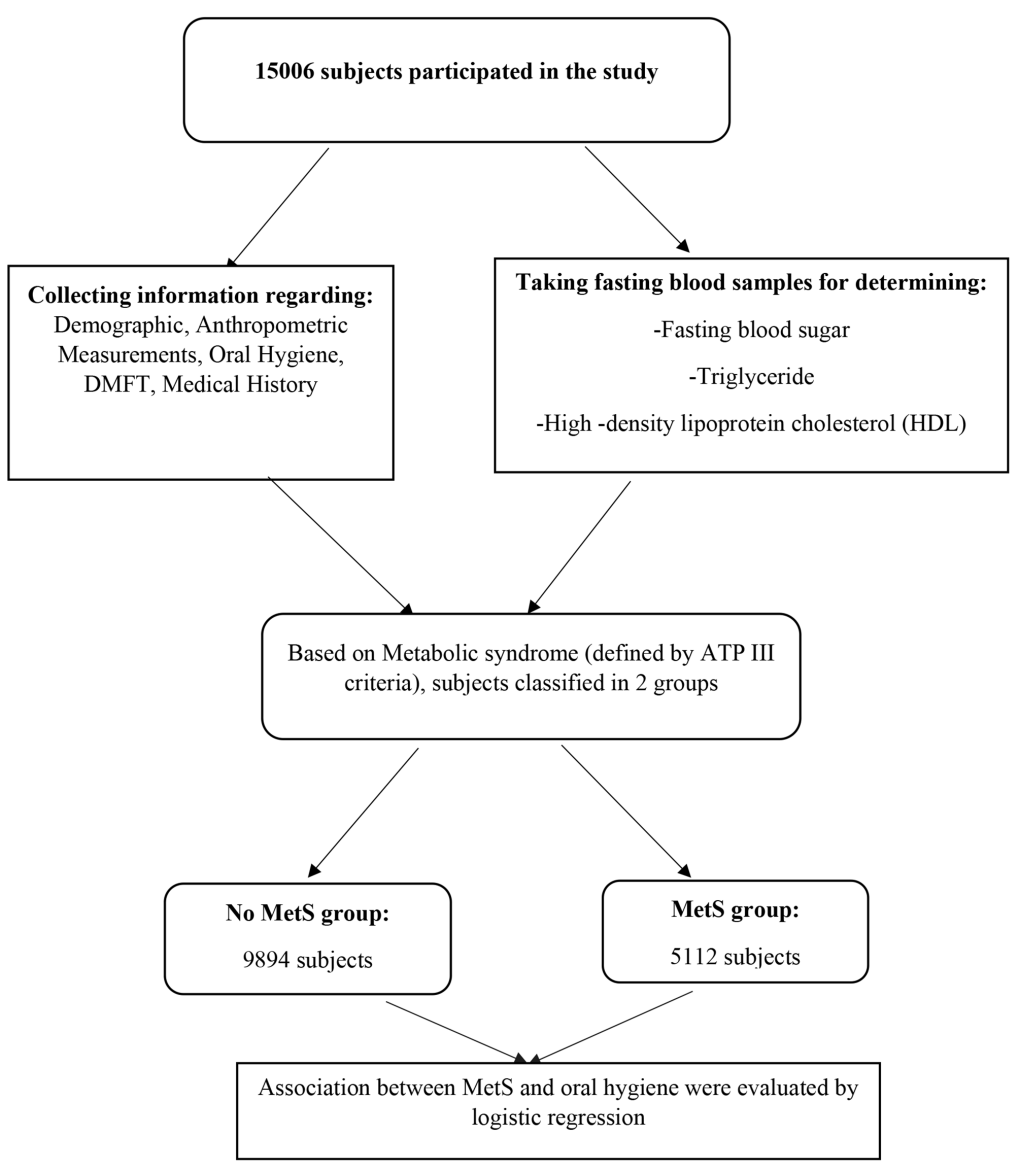
Flow chart of the study

A total of 15,006 participants were included in this study. We divided the participants to two groups based on the presence or absence of MetS (Fig. 1). Participants with MetS were called the MetS group, and participants without MetS were called the no MetS group.

### Variables/measurement

#### Metabolic syndrome assessment

Participants were instructed to refer to the cohort center while fasting. After taking fasting blood samples, biochemical factors such as fasting blood sugar (FBS), triglyceride (TG), and high-density lipoprotein (HDL) levels were measured with Parsazmoon kits. The definition of MetS was based on the National Cholesterol Education Program Adult Treatment Panel III (ATP III) criteria [24]. At least three of the following were needed to diagnose MetS: TG ≥ 150 mg/dl (or drug treatment for elevated TG); waist circumference (WC) ≥ 102 cm in men and ≥ 88 cm in women; HDL-C values of < 40 mg/dl in men and < 50 mg/dl in women; systolic blood pressure ≥ 130 mmHg or diastolic blood pressure ≥ 85 mmHg or use of antihypertensive medications; and FBS ≥ 100 mg/dl or use of glucose-lowering drugs.

#### Oral ***hygiene*** assessment

Demographic and oral health information was collected using a questionnaire. Brushing frequency (once a day, twice a day, three times a day, other and not brushing), the Decayed, Missing, and Filled Teeth (DMFT) index, use of dental floss and mouthwash were evaluated.

#### Anthropometric measures

The height, weight, and waist circumference (WC) parameters were measured based on standard criteria. Body mass index (BMI) was calculated using the weight (kg)/height (m^2^) formula.

#### Socioeconomic status (SES) assessment

The SES index was calculated based on the number of trips abroad, access to computers, laptops, washing machines, dishwashers, etc., and the place of residence as a wealth score index (WSI).

### Statistical analysis

Participants were categorized by the presence of MetS, and an appropriate comparative analysis was conducted to determine the differences. In each group, the percentage of each oral health indicator was compared using the chi-square test. Scale variables were compared with the Independent T-test and Mann-Whitney U test considering the data were not normally distributed. The association between oral health behaviors, MetS and its components were evaluated by logistic regression. Confounding factors include age, gender, level of education, and WSI, and models were adjusted for these factors. P < 0.05 indicated statistical significance, and SPSS v. 11(SPSS Inc., Chicago, IL, USA) was used for all analyses.

## Results

### General characteristics of participants

Mean age was 48.28 ± 9.11 years in the no MetS group and 52.29 ± 9.02 years in the MetS group (P < 0.001). The majority of the MetS group (66.4%), and about half (49.5%) of the no MetS group were females (P < 0.001) (Table 1).

**Table 1:** General characteristics of participants stratified by MetS

	MetS		P -value
	No (n = 9894)	Yes (n = 5112)	
	**N(%)**	**N(%)**	
**Gender**			*<0.001
Male	4992(50%)	1720(33.6%)	
Female	4902(49.5%)	3392(66.4%)	
**Education level**			*<0.001
Illiterate	1300(13.1%)	1204(23.6%)	
Primary school	3818(38.6%)	2043(40%)	
Diploma	3780(38.2%)	1556(30.4%)	
University	996(10.1%)	309(6%)	
**Quintiles of wealth index**			< 0.001
1(poorest)	2183(22.1)	1297(25.4)	
2	1578(15.9)	953(18.6)	
3	2033(20.5)	1023(20)	
4	2176(22)	949(18.6)	
45 (richest)	1924(19.4)	890(17.4)	
**Current smoking status**			*<0.001
No smoker	7262(73.4%)	4139(81%)	
Ex-Smoker	852(8.6%)	394(7.7%)	
Smoker	1582(16%)	497(9.7%)	
Smoker other tobacco products (water pipe, hookah,pipe,.)	198(2%)	82(1.6%)	
	**Mean ± SD**	**Mean ± SD**	
**Age(years)**	48.28 ± 9.11	52.29 ± 9.02	**<0.001
**Weight (kg)**	73.14 ± 12.81	81.23 ± 13.70	**<0.001
**Waist circumference (cm)**	90.8 ± 10.56	101.08 ± 9.55	**<0.001
**BMI (kg/m** ^**2**^ **)**	27.45 ± 4.57	31.51 ± 4.47	**<0.001

*P:chi-square test;** P:independent t test

### DMFT and oral hygiene

As shown in the Table 2 the DMFT index in the MetS group was higher than the no MetS group (22.15 ± 8.89 vs. 20.81 ± 8.94) (P < 0.001).

**Table 2:** DMFT and its components and oral health behaviors of participants based on MetS

	MetS		*P-value
	No (n = 9894)	Yes (n = 5112)	
	**Mean ± SD**	**Mean ± SD**	
**DMFT** Median (Min- Max)	20.81 ± 8.94 20 (0–32)	22.15 ± 8.89 22 (0–32)	**<0.001
**Teeth number** Median (Min- Max)	15.37 ± 11.0 19(0–32)	13.35 ± 11.17 16(0–32)	**<0.001
**DMFT components**			
**Decayed Teeth** Median (Min- Max)	2.30 ± 3.76 0(0–32)	2.04 ± 3.40 0(0–32)	**<0.001
**Missing Teeth** Median (Min- Max)	16.08 ± 11.18 13(0–32)	18.13 ± 11.30 15(0–32)	**<0.001
**Filled Teeth** Median (Min- Max)	2.44 ± 3.74 0(0–29)	1.99 ± 3.55 0(0–25)	**<0.001
**Frequency of tooth brushing**			***<0.001
**At least once a day**	3156(31.9)	1359(26.6)	
**Unregularly**	1790(18.1)	872(17.1)	
**Has denture**	3227(32.6)	2049(40.1)	
**Do not brush**	1721(17.4)	832(16.3)	
**Using mouth wash**			***0.47
**NO**	9624(97.3)	4974(97.3)	
**Yes**	270(2.7)	138(2.7)	
**Use of dental floss**			***0.03
**At least once a day**	777(44.1)	302(40.1)	
**Less than once a day**	9117(92.1)	4810(94.1)	

** Mann-Whitney U test; ***P chi-square test

In the MetS group, the frequency of brushing (P < 0.001) and flossing (P = 0.03) was lower than those in the no MetS group, and many of these patients had dentures. Interestingly, 97.3% of the patients never used mouthwash. There was not a significant difference between the two groups in the MetS of using mouthwash (P = 0.47).

Individuals who had brushed their teeth at least once a day had a significantly smaller WC (*p* < 0.001), lower weight (P < 0.01), lower diastolic blood pressure (P < 0.001), lower serum TG (P < 0.001), higher serum HDL-C (P < 0.001), and lower FBS (P < 0.001) (Table 3).

**Table 3:** Anthropometric measurements and biochemical factors of participants in relation to oral health behavior

	Frequency tooth brushing (times/day)					Use of dental floss		
	**At least once a day**	**Unregularly**	**Has denture**	**Do not brush**	*P -value	**At least once a day**	**Less than once a day**	**P -value
	mean ± SD	mean ± SD	mean ± SD	mean ± SD		mean ± SD	mean ± SD	
**Weight (kg)**	75.37 ± 12.98	78.91 ± 13.70	73.40 ± 13.30	78.85 ± 14.62	< 0.001	75.50 ± 12.87	75.93 ± 13.77	0.31
**Hip circumference (cm)**	105.06 ± 8.45	105.76 ± 8.69	103.24 ± 8.85	104.69 ± 9.03	< 0.001	104.79 ± 8.13	104.46 ± 8.84	0.22
**Waist circumference (cm)**	92.44 ± 10.72	95.42 ± 11.06	94.63 ± 11.52	95.69 ± 11.84	< 0.001	92.12 ± 10.42	94.46 ± 11.38	< 0.001
**BMI (kg/m** ^**2**^ **)**	28.66 ± 4.60	29.42 ± 4.88	28.58 ± 5.03	29.04 ± 5.24	< 0.001	28.29 ± 4.48	28.87 ± 4.95	< 0.001
**SBP (mmHg)**	110.35 ± 15.91	111.99 ± 16.01	116.71 ± 17.94	115.34 ± 17.15	< 0.001	108.30 ± 14.38	114.15 ± 17.22	< 0.001
**DBP (mmHg)**	72.45 ± 9.67	73.40 ± 9.71	73.77 ± 9.67	74.66 ± 10.06	< 0.001	71.68 ± 9.30	73.60 ± 9.80	< 0.001
**Cholesterol (mg/dl)**	190.01 ± 38.25	191.53 ± 39.80	197.97 ± 41.42	189.20 ± 39.94	< 0.001	189.08 ± 38.75	193.24 ± 40.21	0.001
**TG(mg/dl)**	146.81 ± 78.28	157.07 ± 86.10	148.09 ± 83.25	153.62 ± 95.84	< 0.001	148.63 ± 81.29	150.36 ± 84.95	0.51
**FBS (mg/dl)**	93.87 ± 26.40	97.53 ± 30.22	103.23 ± 35.81	101.52 ± 35.08	< 0.001	91.77 ± 25.05	99.68 ± 32.79	< 0.001
**HDL-C(mg/dl)**	46.06 ± 10.60	44.96 ± 10.47	47.38 ± 11.44	44.16 ± 10.51	< 0.001	45.88 ± 11.02	46.02 ± 10.92	0.68
**LDL (mg/dl)**	114.56 ± 32.66	115.28 ± 33.94	121.07 ± 35.37	114.45 ± 34.43	< 0.001	113.73 ± 33.60	117.20 ± 34.33	0.001

*P: ANOVA; ** P: independent t test

Participants who flossed at least once a day had a significantly smaller WC (P < 0.001), lower BMI (P < 0.001), lower serum Cholesterol (P = 0.001), lower SBP (P < 0.001), lower DBP (P < 0.001), lower serum LDL-C (P = 0.001), and lower FBS (P < 0.001) (Table 3).

### Oral and dental risk factors of MetS

**Fig. 2:**
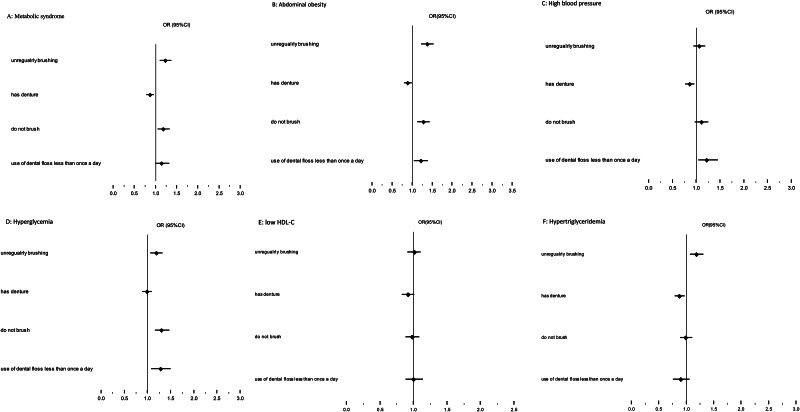
The association between oral health behaviors of participants and MetS and its components (adjusted for age, gender, education level, socioeconomic status)

A logistic regression model was used to identify the associations between oral health behaviors and MetS. An increasing crude odds of MetS (unadjusted OR = 1.47, 95%CI = 1.35–1.60), abdominal obesity (unadjusted OR = 1.16, 95%CI = 1.07–1.25), high blood pressure (unadjusted OR = 2.25, 95%CI = 2.06–2.46), and hyperglycemia (unadjusted OR = 2.00, 95%CI = 1.82–2.18) were observed in subjects who had a denture (Table 4). After adjusting for age and gender, education level, and WSI having a denture became a protective factor (Fig. 2).

**Table 4:** The association between oral health behaviors of participants and MetS and its components

	frequency of tooth brushing				Use of dental floss	
	At least once a day	Unregularly	Has denture	Do not brush	At least once a day	Less than once a day
	OR (95%CI)	OR (95%CI)	OR (95%CI)	OR (95%CI)	OR (95%CI)	OR (95%CI)
**MetS**						
Unadjusted	Reference	1.13(1.02–1.25)	1.47(1.35–1.60)	1.12(1.01–1.24)	Reference	1.35(1.18–1.55)
**Abdominal obesity**						
Unadjusted	Reference	1.00(0.91–1.10)	1.16(1.07–1.25)	0.77(0.70–0.85)	Reference	1.17(1.03–1.32)
**High blood pressure**						
Unadjusted	Reference	1.09(0.97–1.22)	2.25(2.06–2.46)	1.40(1.25–1.56)	Reference	1.86(1.60–2.17)
**Hyperglycemia**						
Unadjusted	Reference	1.28(1.15–1.43)	2.00(1.82–2.18)	1.69(1.52–1.89)	Reference	1.88(1.61–2.20)
**Low HDL-c**						
Unadjusted	Reference	0.89(0.81–0.98)	0.72(0.66–0.78)	0.75(0.68–0.82)	Reference	0.82(0.73–0.93)
**Hypertriglyceridemia**						
Unadjusted	Reference	1.19(1.08–1.31)	0.97(0.89–1.05)	1.02(0.92–1.13)	Reference	0.89(0.78–1.01)

Not brushing at all was predictive of increased odds of MetS (unadjusted OR = 1.12, 95%CI = 1.01–1.24), high blood pressure (unadjusted OR = 1.40, 95%CI = 1.25–1.56), and hyperglycemia (unadjusted OR = 1.69, 95%CI = 1.52–1.89) (Table 4). After adjusting, not brushing the teeth was predictive of the odds of MetS (OR = 1.18, 95%CI = 1.05–1.33), abdominal obesity (OR = 1.28, 95%CI = 1.13–1.44), and hyperglycemia (OR = 1.30, 95%CI = 1.16–1.47) (Fig. 2).

Irregular brushing increased the crude odds ratio of MetS by 1.13 folds (unadjusted OR = 1.13, 95%CI = 1.02–1.25), hyperglycemia (unadjusted OR = 1.28, 95%CI = 1.15–1.43), and hypertriglyceridemia (unadjusted OR = 1.19, 95%CI = 1.08–1.31) (Table 4). After adjusting for confounding factors, irregular brushing was associated with increased odds of MetS (OR = 1.23, 95%CI = 1.10–1.37), abdominal obesity (OR = 1.38, 95%CI = 1.23–1.54), hypertriglyceridemia (OR = 1.18, 95%CI = 1.07–1.31), and hyperglycemia (OR = 1.19, 95%CI = 1.06–1.33) (Fig. 2)

Flossing less than once a day increased the odds of abdominal obesity (unadjusted OR = 1.17, 95%CI = 1.03–1.32) and hyperglycemia (unadjusted OR = 1.88, 95%CI = 1.61–2.20) (Table 4) when unadjusted; after adjustment for age, gender, education, and WSI, the increase was still significant (Fig. 2).

## Discussion

The prevalence of MetS in our study was 34.06%, which is similar to the study of Souza et al. [25] and Kim et al. [26, 27] and higher compared to Kobayashi et al. [27] and Furuta et al. [28] studies. Habitual similarities in different societies could explain the differences in MetS prevalence. The lower prevalence of MetS in latter studies may be due to the higher SES of their participants.

Our findings suggest that in people who do not brush their teeth, there is an increased risk of abdominal obesity, and hyperglycemia after adjustment for age, gender, education, and WSI, and therefore the risk of MetS was higher in those people. We found out that irregular tooth brushing was associated with increased odds of MetS, abdominal obesity, hypertension, hyperglycemia, and hypertriglyceridemia after adjusting for confounding factors; these findings are consistent with Furuta et al., [28] which was carried out as a 5-year longitudinal study and established that tooth brushing habits might play a role in obesity and hyperglycemia. Kobayashi et al. [27], evaluated whether brushing the teeth more frequently can prevent MetS and found that brushing the teeth more than three times a day has a protective effect against MetS and hypertriglyceridemia. Tooth brushing leads to a reduction in oral bacteria and also lower inflammation, which is associated with higher MetS prevalence in former studies [26].

As mentioned before, several studies suggest that dysbiosis of oral microbiota is responsible for such results, and breaking the vicious cycle of dysbiosis could further enhance the metabolic state [29–32].

Having a denture was associated with increased crude odds of MetS, abdominal obesity, hypertension, and hyperglycemia; after adjustment for age, gender, education, and WSI, interestingly, having a denture was associated with decreased odds of MetS, abdominal obesity, hypertension, and hypertriglyceridemia; which supports the theory of mild chronic inflammation as a cause for MetS; as in the study of Khazaei et al. [33] in Iran, the prevalence of diabetes was lower among patients with edentulism. Teles et al. [34] found out that after a professional cleaning, dentures will have lesser colonization of total bacteria compared to natural teeth. In a comparative study, O’Donnell et al. [35] explored the composition of denture plaque and pointed out that in edentulous patients the microbiome has less diversity and less colonization of *Actinomyces, Haemophilus, Corynebacterium*, and *Veillonella* compared to subjects who had both the denture and natural teeth. Our study is consistent with these results as we also demonstrated that having dentures might have a protective effect against MetS by reducing dysbiosis.

However, many studies argued that edentulism is associated with increased odds of MetS and its components; Zhu et al. [36] found out teeth number had a reverse relation with MetS and its components although this association was not observed in older adults. The difference may be the result of undiagnosed cofounder factors related to the age of participants.

We also observed that participants with MetS lost more teeth than the general population, similar to Souza et al. [25], Ono et al. [37], and Saito et al. [38] studies. These might be caused by similar oral health routines and higher consumption of fermentable carbohydrates and/or high-fat foods in the mentioned studies. These findings may be initially confusing; however, it is suspected that losing teeth in MetS participants results from inflammation. Follow-up and comparing the oral microbiota of the patients and inflammatory markers could enlighten this matter.

In our study mean DMFT index was 22.15 ± 8.89 in the MetS group and 20.81 ± 8.94 in the no MetS group. The relationship between DMFT and MetS in this study was similar to previous studies; however, the DMFT index was much more than the study of Adachi et al.,[39] and Iwasaki et al. [40]. This difference may be attributed to higher health awareness among the participants of those studies. The higher mean missing teeth number in our study can indicate lower oral hygiene and a lack of routine health check-ups in our population.

Unlike the study of Kim et al. [26], in our population, not using dental floss was only associated with abdominal obesity and hyperglycemia. In contrast, they found that not using dental floss was associated with MetS, and hypertension, and was not associated with hyperglycemia. This inconsistency may be due to infrequent flossing among participants of our study. This is the first study to assess the correlation of oral health behaviors with MetS and its components in Iran. The large sample size and inclusion of different population groups, make the results more generalizable. However, this study had two limitations: First, the associations were not adjusted for dietary habits and periodontitis. Second, this was a MetS -no MetS study, and further research such as animal experiments and cohort follow-up studies could lead to clear findings.

In conclusion, oral hygiene behavior is associated with the components of MetS. As observed in our study, the rate of MetS and dental caries is higher and oral hygiene is worse in the MetS group. Further studies are suggested to encourage oral hygiene among the general population which has more beneficiary effects than has been known before.

## Data Availability

All collected data analyzed during this study are included in this published article. The datasets used and/or analyzed during the current study are available from the corresponding author (Elnaz Faramarzi;email:elnazfaramarzi849@gmail.com) on reasonable request. The data are not publicly available due to privacy or ethical restrictions.

## References

[1] Kamiab N, Kamalabadi YM, Fathollahi MS (2021). DMFT of the First Permanent Molars, dmft and related factors among all First-Grade Primary School students in Rafsanjan Urban Area. J Dent.

[2] Kassebaum N, Bernabé E, Dahiya M, Bhandari B, Murray C, Marcenes W (2015). Global burden of untreated caries: a systematic review and metaregression. J Dent Res.

[3] Moradi G, Bolbanabad AM, Moinafshar A, Adabi H, Sharafi M, Zareie B (2019). Evaluation of oral health status based on the decayed, missing and filled teeth (DMFT) index. Iran J Public Health.

[4] Chapple I, Genco R (2013). Working group 2 of the joint EFP/AAP workshop. Diabetes and periodontal diseases: consensus report of the Joint EFP. AAP Workshop on periodontitis and systemic diseases J Periodontol.

[5] Nibali L, Tatarakis N, Needleman I, Tu Y-K, D’Aiuto F, Rizzo M, Donos N (2013). Association between metabolic syndrome and periodontitis: a systematic review and meta-analysis. J Clin Endocrinol Metab.

[6] Tonetti MS, Van Dyke TE (2013). Periodontitis and atherosclerotic cardiovascular disease: consensus report of the joint EFP/AAP workshop on Periodontitis and systemic Diseases. J Periodontol.

[7] Barrington G, Khan S, Kent K, Brennan DS, Crocombe LA, Bettiol S (2019). Obesity, dietary sugar and dental caries in australian adults. Int Dent J.

[8] Gobin R, Tian D, Liu Q, Wang J. Periodontal diseases and the risk of metabolic syndrome: an updated systematic review and meta-analysis. Front Endocrinol 2020;11:336. doi: 10.3389/fendo.2020.00336. 10.3389/fendo.2020.00336 PMC7296056 32582028

[9] Abdalla-Aslan R, Findler M, Levin L, Zini A, Shay B, Twig G, Almoznino G (2019). Where periodontitis meets metabolic syndrome—the role of common health‐related risk factors. J Oral Rehabil.

[10] Sigit FS, Tahapary DL, Trompet S, Sartono E, van Willems K, Rosendaal FR, De Mutsert R (2020). The prevalence of metabolic syndrome and its association with body fat distribution in middle-aged individuals from Indonesia and the Netherlands: a cross-sectional analysis of two population-based studies. Diabetol Metab Syndr.

[11] Solomon S, Mulugeta W (2019). Disease burden and associated risk factors for metabolic syndrome among adults in Ethiopia. BMC Cardiovasc Disord.

[12] Fatahi A, Doosti-Irani A, Cheraghi Z (2020). Prevalence and incidence of metabolic syndrome in Iran: a systematic review and meta-analysis. Int J Prev Med.

[13] Saklayen MG. The global epidemic of the metabolic syndrome. Curr Hypertens Rep. 2018 F;20(2):12. doi: 10.1007/s11906-018-0812-z. 10.1007/s11906-018-0812-z PMC5866840 29480368

[14] Marcaccini AM, Meschiari CA, Sorgi CA, Saraiva MC, de Souza AM, Faccioli LH (2009). etal.Circulating interleukin-6 and high‐sensitivity C‐reactive protein decrease after periodontal therapy in otherwise healthy subjects. J Periodontol.

[15] Kirilmaz B, Asgun F, Alioglu E, Ercan E, Tengiz I, Turk U (2010). High inflammatory activity related to the number of metabolic syndrome components. J Clin Hypertens.

[16] Si J, Lee C, Ko G (2017). Oral microbiota: microbial biomarkers of metabolic syndrome independent of host genetic factors. Front Cell Infect Microbiol.

[17] LaMonte MJ, Williams AM, Genco RJ, Andrews CA, Hovey KM, Millen AE (2014). Association between metabolic syndrome and periodontal disease measures in postmenopausal women: the Buffalo OsteoPerio study. J periodontol.

[18] Nibali L, Donos N, Terranova V, Di Pino A, Di Marca S, Ferrara V (2019). Left ventricular geometry and periodontitis in patients with the metabolic syndrome. Clin Oral Invest.

[19] Rimondini L, Zolfanelli B, Bernardi F, Bez C (2001). Self-preventive oral behavior in an italian university student population. J Clin Periodontol.

[20] Tahamtan S, Shirban F, Bagherniya M, Johnston TP, Sahebkar A (2020). The effects of statins on dental and oral health: a review of preclinical and clinical studies. J Transl Med.

[21] Hamasha AA-H, Aldosari MN, Alturki AM, Aljohani SA, Aljabali IF, Alotibi R (2019). Barrier to access and dental care utilization behavior with related independent variables in the elderly population of Saudi Arabia. J Int Soc Prev Community Dent.

[22] Poustchi H, Eghtesad S, Kamangar F, Etemadi A, Keshtkar A-A, Hekmatdoost A (2018). Prospective epidemiological research studies in Iran (the PERSIAN Cohort Study): rationale, objectives, and design. Am J Epidemiol.

[23] Farhang S, Faramarzi E, Amini Sani N, Poustchi H, Ostadrahimi A, Alizadeh BZ, Somi MH (2019). Cohort profile: the AZAR cohort, a health-oriented research model in areas of major environmental change in Central Asia. Inter J Epidemiol.

[24] Alberti KG, Eckel RH, Grundy SM, Zimmet PZ, Cleeman JI, Donato KA (2009). etal.Harmonizing the metabolic syndrome: a joint interim statement of the international diabetes federation task force on epidemiology and prevention; national heart, lung, and blood institute; american heart association; world heart federation; international atherosclerosis society; and international association for the study of obesity. Circulation.

[25] Souza ML, Nascimento GG, González-Chica DA, Peres KG, Peres MA (2022). Counterfactual approach on the effect of metabolic syndrome on tooth loss: a population‐based study. J Periodontol.

[26] Kim Y-H, Kim D-H, Lim KS, Ko B-J, Han B-D, Nam G-E (2014). Oral health behaviors and metabolic syndrome: the 2008–2010 korean National Health and Nutrition Examination Survey. Clin Oral Invest.

[27] Kobayashi Y, Niu K, Guan L, Momma H, Guo H, Cui Y, Nagatomi R (2012). Oral health behavior and metabolic syndrome and its components in adults. J Dent Res.

[28] Furuta M, Takeuchi K, Takeshita T, Tanaka A, Suma S, Shinagawa T (2020). Longitudinal associations of toothbrushing with obesity and hyperglycemia. J Epidemiol.

[29] Isacco CG, Ballini A, De Vito D, Nguyen KC, Cantore S, Bottalico L (2021). Rebalancing the oral microbiota as an efficient tool in endocrine, metabolic and immune disorders. Endocr Metab Immune Disord Drug Targets.

[30] Minty M, Canceil T, Serino M, Burcelin R, Tercé F, Blasco-Baque V (2019). Oral microbiota-induced periodontitis: a new risk factor of metabolic diseases. Rev Endocr Metab Disord.

[31] Latti BR, Kalburge JV, Birajdar SB, Latti RG (2018). Evaluation of relationship between dental caries, diabetes mellitus and oral microbiota in diabetics. J Oral Maxillofac Pathol.

[32] Lamont RJ, Koo H, Hajishengallis G (2018). The oral microbiota: dynamic communities and host interactions. Nat Rev Microbiol.

[33] Khazaei S, Keshteli H, Feizi A, Savabi A, Adibi O. P. Epidemiology and risk factors of tooth loss among Iranian adults: findings from a large community-based study. BioMed Res Inter. 2013; 2013. 10.1155/2013/786462. 10.1155/2013/786462 PMC3818816 24228259

[34] Teles FR, Teles R, Sachdeo A, Uzel N, Song X, Torresyap G (2012). Comparison of microbial changes in early redeveloping biofilms on natural teeth and dentures. J Periodontol.

[35] O’Donnell LE, Robertson D, Nile CJ, Cross LJ, Riggio M, Sherriff A, Bradshaw D, Lambert M, Malcolm J, Buijs MJ (2015). The oral microbiome of denture wearers is influenced by levels of natural dentition. PLoS ONE.

[36] Zhu Y, Hollis JH (2015). Associations between the number of natural teeth and metabolic syndrome in adults. J Clin Periodontol.

[37] Ono T, Kato S, Kokubo Y, Hasegawa Y, Kosaka T, Maeda Y et al. Tooth Loss Related with Prevalence of Metabolic Syndrome in a General Urban Japanese Population: The Suita Study. International Journal of Environmental Research and Public Health 2022, 19(11):6441. Int J Environ Res Public Health. 2022;19(11):6441. doi: 10.3390/ijerph19116441. 10.3390/ijerph19116441 PMC9180197 35682027

[38] Saito M, Shimazaki Y, Nonoyama T, Tadokoro Y. Number of teeth, oral self-care, eating speed, and metabolic syndrome in an aged Japanese population. Journal of epidemiology 2019, 29(1):26–32. Epidemiol. 2019;29(1):26–32. doi: 10.2188/jea.JE20170210. 10.2188/jea.JE20170210 PMC6290276 29910228

[39] Adachi N, Kobayashi Y (2020). One-year follow-up study on associations between dental caries, periodontitis, and metabolic syndrome. J Oral Sci.

[40] Iwasaki T, Hirose A, Azuma T, Ohashi T, Watanabe K, Obora A, Deguchi F, Kojima T, Isozaki A, Tomofuji T (2019). Associations between caries experience, dietary habits, and metabolic syndrome in japanese adults. J Oral Sci.

